# Bone density as a marker for local response to radiotherapy of spinal bone metastases in women with breast cancer: a retrospective analysis

**DOI:** 10.1186/s13014-015-0368-x

**Published:** 2015-03-07

**Authors:** Robert Foerster, Christian Eisele, Thomas Bruckner, Tilman Bostel, Ingmar Schlampp, Robert Wolf, Juergen Debus, Harald Rief

**Affiliations:** Department of Radiation Oncology, University Hospital Heidelberg, Im Neuenheimer Feld 400, 69120 Heidelberg, Germany; Department of Medical Biometry, University Hospital Heidelberg, Im Neuenheimer Feld 305, 69120 Heidelberg, Germany

**Keywords:** Bone density, Bone metastases, Breast cancer, Radiotherapy, Local response

## Abstract

**Background:**

We designed this study to quantify the effects of radiotherapy (RT) on bone density as a local response in spinal bone metastases of women with breast cancer and, secondly, to establish bone density as an accurate and reproducible marker for assessment of local response to RT in spinal bone metastases.

**Methods:**

We retrospectively assessed 135 osteolytic spinal metastases in 115 women with metastatic breast cancer treated at our department between January 2000 and January 2012. Primary endpoint was to compare bone density in the bone metastases before, 3 months after and 6 months after RT. Bone density was measured in Hounsfield units (HU) in computed tomography scans. We calculated mean values in HU and the standard deviation (SD) as a measurement of bone density before, 3 months and 6 months after RT. *T*-test was used for statistical analysis of difference in bone density as well as for univariate analysis of prognostic factors for difference in bone density 3 and 6 months after RT.

**Results:**

Mean bone density was 194.8 HU ± SD 123.0 at baseline. Bone density increased significantly by a mean of 145.8 HU ± SD 139.4 after 3 months (p = .0001) and by 250.3 HU ± SD 147.1 after 6 months (p < .0001). Women receiving bisphosphonates showed a tendency towards higher increase in bone density in the metastases after 3 months (152.6 HU ± SD 141.9 vs. 76.0 HU ± SD 86.1; p = .069) and pathological fractures before RT were associated with a significantly higher increase in bone density after 3 months (202.3 HU ± SD 161.9 vs. 130.3 HU ± SD 129.2; p = .013). Concomitant chemotherapy (ChT) or endocrine therapy (ET), hormone receptor status, performance score, applied overall RT dose and prescription of a surgical corset did not correlate with a difference in bone density after RT.

**Conclusions:**

Bone density measurement in HU is a practicable and reproducible method for assessment of local RT response in osteolytic metastases in breast cancer. Our analysis demonstrated an excellent local response within metastases after palliative RT.

## Background

The bone is the most common site for metastases in women with breast cancer [1]. Bone metastases of the spinal column are a major cause of morbidity and reduced quality of life due to severe pain, pathological fractures, spinal cord compression and hypercalcemia [2,3]. Bone metastases require a multimodal treatment approach including radiotherapy (RT), minimal invasive surgery and systemic treatments such as bisphosphonates [4]. RT is the most common treatment method [5,6], and its indications are typically pain, instability or neurological symptoms due to spinal cord compression [7]. The simultaneous delivery of RT and bisphosphonates may be beneficial for re-ossification of the bone affected by osseous metastases [8-10]. Previously we were able to show that RT is capable of promoting re-ossification leading to increased stability of spinal bone metastases [11-13]. Secondly, in a recent trial we were able to show that the quantification of bone density within metastases was an accurate and practicable method to evaluate local response after RT [14]. The aim of our current analysis was to quantify the effects of RT on bone density in the metastatic bone in breast cancer patients with spinal bone metastases and to establish bone density as a marker for assessment of local response to RT.

## Methods

**Figure 1:**
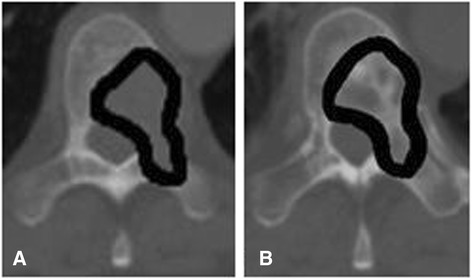
**Osteolytic thoracic spinal metastasis (A) before RT and (B) 3 months after RT as a basis for bone density measurement in HU by manual ROI setting.**

We retrospectively assessed 135 osteolytic metastases of the thoracic and lumbar vertebral column treated with RT at our department between January 2000 and January 2012. The spinal bone metastases were found in 115 women with metastatic breast cancer. Patients’ data were collected from the local cancer registry. Median age was 60 years (range 32–88) and median Karnofsky performance status (KPS) was 80% at first presentation. Seventy-six patients (56.3%) had more than one spinal bone metastasis. Cases characteristics are shown in Table 1. The cases selected for this study were those with available minimum follow-up computed tomography (CT) scans for 3 months after RT. For patients that underwent RT for several regions, each irradiated region was regarded separately as an individual case and in each region only the metastasis with the highest degree of instability according to Taneichi et al. was included in our study [15]. The primary endpoint of this study was to compare bone density in the irradiated metastasis before RT and 3 months as well as 6 months after RT. Additionally we performed a reference measurement of the bone density in the neighboring irradiated vertebral body which was not affected by bone metastases. Most patients were treated additionally with bisphosphonates during RT (91.1%), which represents a major bias for the assessment of treatment response in the metastasis. Therefore, a bone density measurement of uninvolved vertebral bodies was executed to detect the increase by a systemic treatment. Bone density was assessed in Hounsfield units (HU) by manual region of interest (ROI) setting of the whole vertebral body for uninvolved bone and within metastases for involved bone (Figure 1). The study was approved by the university’s ethical committee (# S-513/2012).

**Table 1:** **Cases characteristics**

**Age**		
Median	60 years	
Range	32-88 years	
	**n**	**%**
**Karnofsky performance status**		
30-70%	40	29.6%
80-90%	95	70.3%
**Histology**		
Invasive ductal	107	79.3%
Invasive lobular	28	20.7%
**Receptor status positivity**		
ER (N = 34)	28	82.4%
PgR (N = 50)	40	80.0%
HER2 (N = 135)	39	28.9%
**Site**		
Thoracic	99	73.3%
Lumbar	36	26.7%
**Number of bone metastases**		
Solitary	59	43.7%
Multiple	76	56.3%
**Treatment indications**		
Pain	69	51.1%
Instability	44	32.6%
**Pathological fracture**		
Before RT	29	21.5%
After RT	8	5.9%
**Surgical corset**		
During RT	82	60.7%

### Radiotherapy

RT was planned as virtual simulation based on planning CT imaging and was delivered over a dorsal 6 MV photon filed. The planning target volume (PTV) covered the affected vertebral bodies as well as the ones directly above and below. Median prescribed total dose was 30.0 Gy in 3.0 Gy single fractions. Treatment characteristics are shown in Table 2.

**Table 2:** **Treatment of cases**

	**n**	**%**
**Radiotherapy (RT) (N = 133)**		
10 × 3 Gy	77	57.9%
14 × 2.5 Gy	20	15.1%
20 × 2 Gy	34	25.6%
Others	2	1.5%
**Systemic therapy prior to RT**		
Chemotherapy	46	34.1%
Endocrine therapy	47	34.8%
Bisphosphonates	51	37.8%
**Systemic therapy after RT**		
Chemotherapy	72	53.3%
Endocrine therapy (N = 30)	26	86.7%
Bisphosphonates	123	91.1.%

### Statistical analysis

We calculated mean values in HU and the standard deviation (SD) as a measurement for bone density before as well as 3 and 6 months after RT. Regarding statistical analysis of difference in bone density as well as for univariate analysis of prognostic factors for difference in bone density at 3 and at 6 months after RT we calculated the equality of variances and used the *t*-test. As possible prognostic factors we investigated systemic therapy (chemotherapy (ChT) and endocrine therapy (ET)) before/after RT, bisphosphonates after RT, treatment indications (pain, stability), prescription of a surgical corset, irradiated area (lumbar vs. thoracic), number of metastases (1 vs. >1), prescribed overall RT dose, pathological fractures before/after RT and hormone receptor status (estrogen (ER), progesterone (PgR), Her-2/neu (HER2)). A p-value ≤ 0.05 was considered statistically significant. All statistical analyses were performed with SAS software 9.1 (SAS Institute, Cary, NC, USA).

## Results

The mean calculated size of the metastases was 431.3 mm^2^ ± SD 313.5 and the mean bone density in the metastases was 194.8 HU ± SD 123.0 at initial assessment. Three months after RT we observed a mean bone density of 340 HU ± SD 179.2 and after 6 months a mean bone density of 433.1 HU ± SD 172.6 in the metastases. Whereas mean bone density in the irradiated unaffected neighboring vertebral bodies was 235.9 HU ± SD 143.4 before RT, 228.6 HU ± SD 143.2 after 3 months and 250.3 HU ± SD 147.1 after 6 months. Bone density increased significantly in the metastases during follow-up after RT. At 3 months the bone density had increased by a mean of 145.8 HU ± SD 139.4 (p < .0001) and after 6 months by a mean of 238.0 HU ± SD 149.2 (p < .0001). The bone density in the irradiated unaffected neighboring vertebral bodies used for reference measurements did not change significantly during follow-up after RT. After 3 months we found a slight decrease by a mean of −7.3 HU ± SD 60.4 (p = .162) and after 6 months, with a mean decrease of −0.1 HU ± SD 70.1 (p = .993), there was practically no change in bone density observable (Table 3).

**Table 3:** **Bone density (HU) in metastases and in irradiated uninvolved bone**

	**Mean**	**SD**	**Mean difference**	**SD**	**p-value**
**Bone metastases**					
Before RT	194.8	123.0			
After 3 months	340.6	179.2	145.8	139.4	p < .0001
After 6 months	433.1	172.6	238.0	149.2	p < .0001
**Irradiated uninvolved bone**					
Before RT	235.9	143.4			
After 3 months	228.6	143.2	−7.3	−1.41	p = .162
After 6 months	250.3	147.1	−0.1	−0.01	p = .993

Increase in bone density of the metastases seemed to be associated with the prescription of bisphosphonates. While women receiving bisphosphonates had a mean increase in bone density of 152.59 HU ± SD 141.99 in the metastases after 3 months, patients without bisphosphonates only had a mean increase in bone density of 76.03 HU ± SD 86.6 (p = .069) in the metastases 3 months following RT. Additionally we found that women with pathological fractures before RT (21.5%) had a significantly higher increase in bone density after 3 months than those which presented without fractures at initial assessment (202.3 HU ± SD 161.88 vs. 130.33 HU ± SD 129.23; p = .013). These differences were no longer detectable 6 months after RT. All other investigated potentially prognostic factors, especially concomitant ChT or ET, hormone receptor status, KPS, applied overall dose as well as the prescription of a surgical corset, did not significantly correlate with an increase or decrease in bone density after RT (Table 4).

**Table 4:** **Univariate analysis of prognostic factors for difference in bone density in HU**

	**After 3 months**				**After 6 months**			
	**n**	**Mean**	**SD**	**p-value**	**n**	**Mean**	**SD**	**p-value**
**Bisphosphonates after RT**				p = .069				p = .162
Yes	123	152.6	141.9		76	245.8	151.5	
No	12	76.0	86.1		9	171.9	114.4	
**Pathological fracture before RT**				p = .013				p = .801
Yes	29	202.3	161.9		21	230.8	141.7	
No	106	130.3	129.2		64	240.3	152.6	
**Pathological fracture after RT**				p = .399				p = .399
Yes	8	186.3	133.6		8	280.5	135.3	
No	127	143.2	139.9		77	233.5	150.7	
**Chemotherapy before RT**				p = .946				p = .991
Yes	46	144.7	135.3		23	238.3	172.9	
No	89	146.4	148.6		62	237.8	140.9	
**Chemotherapy after RT**				p = .741				p = .547
Yes	72	149.5	130.9		45	247.2	144.9	
No	63	141.5	149.5		40	227.5	154.9	
**Endocrine therapy before RT**				p = .211				p = .133
Yes	47	125.2	137.3		25	200.2	136.1	
No	88	156.8	140.1		60	253.7	152.6	
**Endocrine therapy after RT**				p = .536				p = .657
Yes	26	121.9	98.1		17	196.3	143.6	
No	4	87.8	125.4		2	250.0	316.8	
**Pain as indication for RT**				p = .822				p = .963
Yes	69	148.5	141.5		46	238.7	143.3	
No	66	143.0	138.3		39	237.1	157.7	
**Instability as indication for RT**				p = .479				p = .554
Yes	91	139.9	133.9		57	231.2	141.9	
No	44	158.1	150.9		28	251.7	164.8	
**Surgical corset**				p = .358				p = .213
Yes	82	136.9	134.9		52	221.8	144.6	
No	53	159.6	146.3		33	263.4	154.9	
**Spine**				p = .437				p = .858
Thoracic	99	150.7	149.1		65	239.6	155.9	
Lumbar	36	132.3	109.2		20	232.7	128.1	
**Number of metastases**				p = .983				p = .382
1	59	146.1	132.4		35	254.9	158.2	
>1	76	145.6	145.5		50	226.1	142.9	
**Overall dose**				p = .886				p = .654
<=30 Gy	79	145.1	130.9		57	229.6	143.8	
>30 Gy	54	141.6	150.8		26	245.6	162.9	
**KPS**				p = .815				p = .412
</=70%	40	150.1	148.1		23	263.4	259.9	
>70%	95	143.9	136.4		62	228.5	163.7	
**Estrogen receptor status**				p = .278				p = .828
Positive	28	139.9	115.2		19	222.5	156.6	
Negative	6	83.8	99.3		2	250.0	316.8	
**Progesterone receptor status**				p = .088				p = .694
Positive	40	159.9	123.5		25	242.7	149.5	
Negative	10	87.8	83.4		4	276.5	214.5	
**Her-2/neu receptor status**				p = .581				p = .379
Positive	39	156.3	118.4		28	220.2	108.2	
Negative	96	141.5	147.5		57	246.7	165.8	

## Discussion

In previous studies we demonstrated that RT is capable of improving stability in spinal bone metastases by facilitating re-ossification [11-14]. With our current analysis we were able to quantify the re-ossification after RT by measuring the change in mean bone density on the basis of x-ray absorption in CT scans and we found that mean bone density, as a local response, increased significantly in the metastases after RT. While mean bone density in the metastases increased by 145.8 HU ± SD 139.4 after 3 months (p < .0001) and by 238.0 HU ± SD 149.2 after 6 months (p < .0001), this was not the case in the irradiated neighboring vertebrae unaffected by bone metastases. Other investigators found bone density to increase after RT as well [16,17]. Currently local response is chiefly assessed by visual judgment of sclerosis of the osteolytic lesions in CT scans, with complete response being classified as complete sclerosis of the metastasis, partial response as >50% regression of the metastasis and no response as an unchanged metastases [18]. Such an evaluation of local treatment response is very subjective and imprecise. We believe bone density measurement to be a more reliable and reproducible method for assessment and quantification of re-ossification as a local response to RT in osteolytic spinal bone metastases.

Clinical and preclinical studies suggest a benefit from combined treatment with systemic bisphosphonates concomitant to RT [19-21] since they have been shown to exhibit cytotoxic and radiosensitizing effects when combined with RT additional to their anti-bone-resorptive properties [22,23]. In our analysis there was a strong tendency towards statistical significance for increased bone density in the metastases with concomitant bisphosphonate treatment 3 months following RT (p = .069) and after 6 months the mean increase in mean bone density was still larger in patients receiving combination treatment compared to those without bisphosphonates (245.8 HU ± SD 151.5 vs. 171.9 HU ± SD 114.4). Nevertheless, the rate of bisphosphonates was high (91.1%). However, this was not the case for the irradiated bone unaffected by metastases. We believe that bisphosphonates may be capable of facilitating RT effects in bone metastases and that these patients may respond more rapidly. Other concomitant systemic treatments in the form of ChT or ET did not affect bone density in our analysis. Similarly, systemic treatment before/after RT did not influence local response in terms of stability in two earlier studies [12,13]. This is probably due to the fact that most patients in our study were already postmenopausal and did received bisphosphonates which was not the case in another previous study where ChT before RT had a negative effect on stability as a local response to RT [11]. Negative effects on bone density by aromatase inhibitors [24] and disturbances in bone remodeling by ChT [25] may have been compensated by concomitant bisphosphonate therapy, tamoxifen treatment probably had a rather bone-protective effect [26] and ChT-associated ovarian failure [27] did not play a relevant role in our cohort.

Furthermore we found that mean bone density increase after 3 months was significantly higher in patients with pathological fractures at initial assessment (p = .013) which can be explained by physiological consolidation processes with callus formation after acute fractures. Nevertheless, pathological fracture may affect the bone density in sintered vertebral body after treatment, but this bias was insignificant small in 5.9% of patients.

We found no statistically significant correlation between the remaining investigated prognostic factors and an increase or decrease in bone density after RT. Koswig and Budach found bone density after 6 months to have increased by 173% after 30 Gy in 10 fractions compared to 120% after a single fraction of 8 Gy [16]. Most women in our analysis were treated with an overall dose of 30 Gy in 3 Gy single fractions and thus we were unable to detect any differences between fractionation schedules. KPS and the prescription of a surgical corset also did not affect response to RT in terms of bone density although a higher KPS and not wearing a surgical corset should in theory be associated with more physical activity which in turn can lead to an improved stability of spinal bone metastases [11,14].

## Conclusions

Bone density increased significantly in the metastases during follow-up after RT, while practically no change was seen in the irradiated bone unaffected by metastases. Bone density measurement in HU is a reliable and reproducible method for assessment of local response in osteolytic metastases after RT. Concomitant bisphosphonate may protect from bone-resorptive effects induced by ChT and ET.
